# Is it me or is it you? Physiological effects of the honey bee microbiota may instead be due to host maturation

**DOI:** 10.1128/mbio.02107-24

**Published:** 2024-09-26

**Authors:** Waldan K. Kwong, Kasie Raymann

**Affiliations:** 1 Instituto Gulbenkian de Ciência, Oeiras, Portugal; 2 Department of Plant and Microbial Biology, North Carolina State University, Raleigh, North Carolina, USA; University of Hawaii at Manoa, Honolulu, Hawaii, USA

**Keywords:** gut microbiota, nonhuman microbiota, entomology, behavior

## Abstract

Microbiota-mediated impacts on host physiology and behavior have been widely reported in honey bees (*Apis mellifera*). However, most of these studies are conducted in artificial lab settings and fail to take into account, or make incorrect assumptions about, the complex physical and social structures inherent to natural hive conditions. A new study by Liberti et al. (J. Liberti, E. T. Frank, T. Kay, L. Kesner, et al., mBio 15:e01034-24, 2024, https://doi.org/10.1128/mbio.01034-24) identifies one such overlooked aspect—the behavioral maturation from nurses to foragers—that can be a serious confounding factor in bee microbiota experiments. Using cuticular hydrocarbon profiling to discern between the two maturation states, they find that multiple physiological and behavioral differences between age-matched lab bees could potentially be explained by their maturation state instead of the intended treatment conditions, such as microbial inoculation. This study serves as a stark wake-up call on the necessity of careful replication and cross-disciplinary knowledge transfer (e.g., between animal specialists and microbiologists) in order to truly understand complex host–microbe systems.

## COMMENTARY

It is now well established that the gut microbiota can make drastic contributions to the metabolism, physiology, and behavior of animal hosts (1). In the case of the honey bee (*Apis mellifera*), recent work has uncovered many potential microbially mediated impacts, including on gut physiology (2), pathogen resistance (3), learning (4, 5), and social interactions (6). Adult worker bees acquire their characteristic microbiota upon emergence from pupation, after which the microbiota rapidly proliferates and fills gut niches within 4–5 days (7). Most studies exploring the contribution of the gut microbiota to various aspects of host physiology are conducted with pupae removed from the hive and emerged sterilely in the lab, as it is easy to simultaneously generate bees with and without their normal microbiota by either exposing or not exposing the new adults to bacteria (e.g., from lab cultures or the gut contents of hive bees). In the lab, cohorts of inoculated or uninoculated adults are then typically kept together in cages of up to several dozen bees and sampled/assayed at time points as required for the experiment.

An underlying assumption of this age-controlled cohort approach is that experimental treatment groups (e.g., with or without microbiota) are directly comparable, without a confounding effect of age or maturation. A new study by Liberti et al. (8) challenges this assumption, showing that co-housed bees at a given time point can exist in two distinct behavioral maturation states: nurses and foragers. Furthermore, they find that the maturation states of individuals within a cage are not independent of each other: the proportion of nurses to foragers within a cage can vary greatly between cages of the same age. In the natural hive environment, the behavioral transition from nurses (who perform in-hive tasks such as brood care) to foragers (who fly outside to collect pollen and nectar) typically occurs 2–3 weeks into adulthood. But an earlier transition is possible, especially in the absence of queen pheromones (9) or older foragers (10, 11), which is likely what happens in lab cages composed solely of young age-matched adult workers.

Nurses and foragers cannot be easily discerned by visual appearance. However, the physiological differences between them are substantial. Foragers tend to have less body fat (12), lower weight (13), atrophied hypopharyngeal glands (14), altered brain structure (15), and distinctive gene expression and DNA methylation patterns (16). Another distinguishing feature between nurses and foragers is in the composition of their cuticular hydrocarbons (CHCs) (17, 18), which play important roles in nestmate recognition and communication. Liberti et al. (8) used gas chromatography/mass spectrometry to categorize caged bees into nurses or foragers based on their CHC profiles, which was how they uncovered the variability in maturation states of co-housed bees.

In light of this finding, the authors reassessed two widely cited claims regarding the impact of the microbiota on bee physiology: that the microbiota increases weight gain (2) and influences CHC profiles (19). With new experiments measuring both endpoint body and gut weights, as well as longitudinal tracking of individual bee body weights over 11 days, they found no significant differences between bees with or without their normal microbiota. Instead, they determined that, independent of colonization status, bees with nurse-like CHC profiles tended to weigh more than those with forager-like CHC profiles; this is consistent with what is known about foragers and nurse bees in natural hive conditions (13). They also found no significant difference in CHC profiles between bees treated with four different gut microbiota inoculums; instead, CHC profiles were segregated according to the typical profiles of foragers or nurses, or transitional states between the two. Finally, the authors reassessed data from their previous behavioral study (6), where they examined the effect of the microbiota on social interaction and foraging behavior in artificial subcolonies consisting of 100 age-controlled worker bees. They found that bees with forager-like CHC profiles at the end of their experiment exhibited more forager-like behavior during the observational phase of the experiment, such as more foraging trips and more time spent in the foraging arena. Intriguingly, they also observed an earlier onset of foraging-like behavior, by ~15 h, of bees with their normal microbiota, suggesting that the microbiota may accelerate the transition from nurse to forager. However, these experiments were conducted in laboratory conditions that only mimic a small portion of the complexity of the natural hive environment. Thus, while this work rightly points out a factor (behavioral maturation) that potentially confounded previous studies, there remain many other underexplored variables that likely impact the translatability between lab experiments and natural conditions.

Honey bee colonies are considered superorganisms, which implies that the biology of individuals cannot be separated from the colony as a whole (20). Therefore, experimentalists should carefully assess how to effectively study honey bee development and behavior under laboratory conditions. For instance, one of the main modulators of worker development, physiology, reproduction, and behavior is the presence of the queen, which emits a “queen signal” that is mainly composed of the queen mandibular pheromone (QMP) (21). Synthetic QMP, which is now commercially available, has been shown to elicit similar biological effects on workers as the presence of a queen (9). To our knowledge, a queen or QMP has not been included in any laboratory studies investigating the impact of the microbiota on honey bee behavior and/or physiology. Thus, the addition of QMP in studies conducted in the laboratory could more accurately reflect natural worker phenotypes and might drastically alter the outcomes of behavioral and physiological responses to different conditions (e.g., microbiota-depleted versus conventional bees).

Aside from focusing on a subset of workers, which represent only a small portion of the individuals within the honey bee superorganism, laboratory studies poorly reflect the physical hive environment, which also plays an important role in dictating worker development and behavior (22, 23). In a typical laboratory bee cage, there is no brood or queen to care for, no comb to build or clean, nowhere to store pollen or nectar, and no, or very minimal, space to “forage.” Does division of labor exist if there is no work to do? Do the physiological designations of a worker and forager (e.g., CHC profiles, weight, and hypopharyngeal gland size) under laboratory conditions accurately correspond to typical worker/forager behavior? Although Liberti et al. (8) created sophisticated automated behavioral-tracking subcolonies that included a nest box and a foraging arena, the nest box did not contain comb or brood and the arena was extremely small (22.5 cm × 13.5 cm). Moreover, it is unclear whether the bees that were observed “foraging” (i.e., visiting the foraging arena) possessed forager CHC profiles or other forager-specific characteristics at the time of the observed behavior; this highlights the need to better assess the links between behavior and physiology.

In order to more precisely study how the microbiota impacts worker biology, future studies should attempt to imitate a more hive-like environment in the lab. For example, behavioral-tracking colonies could include a nest box containing QMP and sterilized comb frames with some drawn comb and grafted larvae and a relatively large flight cage that contains most of the food sources for the colony. Although more difficult to establish, construct, house, and monitor, a setup that includes spaces for the workers to perform caste-associated tasks would more closely mimic the hive environment and natural colony structure. Such an approach would also benefit from closer collaborations between ethologists and microbiologists: it is becoming clear that important aspects of one field could be easily overlooked by members of the other when working in isolation.

Because of their conserved and stable gut microbiota, experimental tractability, and intricate behavior and social structure, honey bees have become an excellent model system for disentangling host–microbe interactions (24). Now, this study by Liberti et al. (8) has demonstrated that their complexity might be a double-edged sword. The authors unexpectedly debunked prominent assumptions about the biology of lab-reared honey bees. This is an extremely important finding that will impact how we design and interpret laboratory studies of host–microbe interactions, particularly in honey bees but potentially also in other social animal models. Aside from the direct implications for the field of host–microbiome research, the results of Liberti et al. (8) emphasize the importance of questioning assumptions, repeating experiments, publishing negative and/or contradictory results, and re-evaluating methodologies and conclusions in the light of new findings. In the words of the science philosopher Karl Popper, “The more we learn about the world … the more conscious, specific, and articulate will be our knowledge of what we do not know, our knowledge of our ignorance” (25).
